# The Registered Nurse Prescriber-Led Triage–Treatment–Continuity Model in Family Medicine: A Practice Innovation and Service Evaluation from Cranston Ridge Medical Clinic

**DOI:** 10.3390/healthcare14131965

**Published:** 2026-07-02

**Authors:** Dawid Karczewski, Tomasz Karczewski, Merjorie M. A. Pinero, Avni K. Patel, Melanie L. Thompson

**Affiliations:** Cranston Ridge Medical Clinic, Calgary, AB T3M 3A9, Canada; tomasz@cranstonridgemedical.com (T.K.); merjorie@cranstonridgemedical.com (M.M.A.P.); avni@cranstonridgemedical.com (A.K.P.); melanie@cranstonridgemedical.com (M.L.T.)

**Keywords:** Registered Nurse Prescriber, family medicine, primary care, triage, urgent care, emergency department diversion, service evaluation, quality improvement, clinical support tools, Alberta, nursing scope of practice

## Abstract

**Highlights:**

**What are the main findings?**

- A single-clinic Registered Nurse Prescriber-led Triage–Treatment–Continuity pathway managed 5032 pathway contacts during a defined 12-month evaluation window, including 5030 stable traffic-light-classified contacts and 2 EMS/911 activations before classification.
- The model combines medical office assistant emergency recognition, RN prescriber stability assessment, traffic-light prioritization, clinical support tools, prescribing and diagnostic ordering within authorized scope, and EMR-supported continuity.

**What are the implications of the main findings?**

- RN prescribers may support same-day and semi-urgent access in family medicine when regulation, training, governance, clinical support tools, and escalation pathways are in place.
- Prospective comparative research should evaluate patient-level outcomes, stakeholder feedback, confirmed emergency department or urgent care diversion, cost-effectiveness, and patient, physician, and nurse satisfaction.

**Abstract:**

**Background/Objectives:** Primary care clinics increasingly receive urgent and semi-urgent requests from patients who may otherwise attend emergency departments or urgent care centres when timely appointments are unavailable. This article describes and evaluates the Cranston Ridge Medical Clinic Registered Nurse Prescriber-led Triage–Treatment–Continuity model in Calgary, Alberta, Canada. **Methods:** The manuscript is reported as a single-clinic practice innovation and service evaluation using aggregate, non-identifying operational data from 1 April 2025 to 31 March 2026. The model combines medical office assistant emergency recognition, RN prescriber-led stability assessment, traffic-light urgency classification, a booking-contingency algorithm, clinical support tools, diagnostic test ordering and prescribing within authorized scope, safety-netting, and communication with the patient’s primary care provider through the electronic medical record. **Results:** During the evaluation period, 5032 pathway contacts were managed. Of 5030 stable contacts assigned traffic-light categories, 4950 (98.4%) were Code Red same-day contacts, 55 (1.1%) were Code Yellow 24–48-h contacts, and 25 (0.5%) were Code Green non-urgent contacts. Two contacts triggered EMS/911 activation before traffic-light classification. Following RN prescriber assessment, 9 emergency department referrals, 2 urgent care referrals, 85 primary care provider follow-up appointments, and 5 patient refusals were recorded; no safety incidents or complaints were recorded in the aggregate monitoring dataset. A CIHI-informed 15% reference scenario corresponds to approximately 755 potentially avoided ED/UCC visits, but no confirmed diversion or monetary savings are claimed. **Conclusions:** The model reframes triage as an integrated primary care intervention that combines assessment, treatment, escalation, and continuity. Further ethics-approved research is required to evaluate patient-level outcomes, safety, confirmed health-system utilization effects, stakeholder experience, and cost-effectiveness.

## 1. Introduction

Emergency departments and urgent care centres are essential components of the healthcare system [1]. However, they are also used by patients whose conditions may be more appropriately addressed in primary care when timely access is available [2,3]. This pattern should not be reduced to patient preference or inappropriate emergency department use. Patients often seek urgent or emergency services because they are symptomatic, anxious, uncertain about the seriousness of their condition, and unable to access same-day or timely primary care [4].

The Canadian Institute for Health Information (CIHI) has developed an indicator for emergency department visits involving conditions that could be managed in primary care. The indicator refers to unscheduled emergency department visits by patients with a primary care sensitive diagnosis who were discharged home and were not triaged as emergent [5]. The CIHI reported that 15% of Canadian emergency department visits between April 2023 and March 2024 were for conditions that could potentially have been managed in primary care, and that more than half of those visits involved conditions potentially manageable virtually [6]. This national estimate is a system-level access benchmark and should not be interpreted as a direct diversion rate for any single clinic-based pathway.

Traditional family medicine booking systems may struggle to distinguish emergent, urgent, semi-urgent, and non-urgent patient needs [7]. Medical office assistants are frequently the first point of contact, but their role is administrative rather than diagnostic. Physicians and nurse practitioners may be fully booked, leaving symptomatic patients with limited options. Telephone advice lines may support navigation, but they may not be able to assess the patient in person, order investigations, prescribe treatment, or document directly in the patient’s primary care electronic medical record (EMR) [8]. Walk-in clinics may improve episodic access, but they can fragment continuity of care [9].

Cranston Ridge Medical Clinic (CRMC) developed a Registered Nurse Prescriber-led Triage–Treatment–Continuity model to address this operational gap. The service was implemented as a clinic-based quality improvement and service management initiative intended to increase timely access, support safe escalation, reduce avoidable external urgent care use, support primary care provider capacity, and preserve continuity within the patient’s primary care medical home.

This model builds on prior work describing the Clinical Nurse Specialist-led management framework at CRMC, in which advanced nursing leadership was positioned as a central mechanism for improving clinic operations, access, and interprofessional workflow [10]. The present article extends that earlier clinic-management work by specifying a replicable RN prescriber-led urgent-access pathway, defining the safety and governance layers that support it, reporting aggregate pathway activity, and clarifying how the activity can and cannot be interpreted as potential emergency department or urgent care diversion.

The aim of this article is to describe the CRMC RN Prescriber-led Triage–Treatment–Continuity model as a practice innovation and service evaluation. Because this was a descriptive service evaluation rather than a hypothesis-testing study, no formal statistical hypothesis was prespecified. The evaluation questions were: how the pathway is structured; what aggregate activity it generated over a defined 12-month period; how the observed activity should be interpreted in relation to possible emergency department or urgent care diversion; and what additional patient-level, stakeholder, safety, and cost data are required for future research.

## 2. Materials and Methods

### 2.1. Design and Service Evaluation Positioning

This manuscript describes a single-clinic practice innovation and service evaluation conducted as part of routine quality improvement, service management, and clinical governance at CRMC. The project was implemented to improve internal access, patient safety, workflow, and continuity of care. It was not designed as a clinical trial, retrospective chart review, comparative effectiveness study, or human-subjects research project, and it did not include a comparator group or inferential statistical testing.

The service evaluation used aggregate, non-identifying operational data only. No patient names, health card numbers, dates of birth, addresses, individual chart notes, individual diagnostic test results, patient quotations, interviews, surveys, biological samples, or experimental interventions were used. No linked patient-level emergency department, urgent care, pharmacy, laboratory, or external administrative datasets were used for this manuscript.

This positioning is consistent with TCPS 2 Article 2.5, which states that quality assurance, quality improvement, program evaluation, performance review, or testing within normal educational requirements, when used exclusively for assessment, management, or improvement purposes, do not constitute research for the purposes of the policy and do not fall within Research Ethics Board review [11]. TCPS 2 also notes that if such data are later proposed for research purposes, secondary use of information may require Research Ethics Board review [11].

The Alberta Health Information Act governs health information in the custody or control of custodians and balances privacy protection with appropriate information sharing to provide health services and manage the health system [12]. In keeping with this framework, the present article uses only aggregate, non-identifying operational data. The ARECCI Ethics Screening Tool was completed for the project and retained as part of internal clinic documentation [13].

### 2.2. Setting

CRMC is a family medicine clinic located in Calgary, Alberta, Canada. The clinic provides primary care services through an interprofessional team that includes two physicians, one nurse practitioner, three registered nurse prescribers, four medical office assistants, and collaborative pharmacy support when appropriate. During the evaluation period, the pathway was available during regular weekday clinic operations and telephone access hours; it was not a 24-h, 7-day-a-week service. Telephone entry into the pathway was available approximately 44.5 h per week, or approximately 2314 h per year before statutory holidays and temporary closures. On-site clinic operating hours were approximately 50 h per week, or approximately 2600 h per year before statutory holidays and temporary closures.

The model was designed around six service goals: early identification of immediately life-threatening presentations; rapid escalation of unstable patients to emergency medical services or the emergency department; same-day assessment of stable urgent patients; 24–48-h assessment of semi-urgent patients; routine primary care provider access for non-urgent patients; and continuity through documentation of RN prescriber assessments and plans in the patient’s EMR. The pathway served registered clinic patients across the lifespan, including adults and children. Pediatric, pregnancy, and prenatal concerns could enter the booking pathway, but RN prescriber management depended on stability, authorized clinical practice area, approved clinical support tools, competence, and availability of physician, nurse practitioner, or emergency escalation when required. The evaluation did not extract the total registered patient panel or total clinic call denominator; this limits interpretation of pathway volume as a rate.

### 2.3. Regulatory and Practice Context

The College of Registered Nurses of Alberta (CRNA) defines the registered nurse scope of practice as the interventions that registered nurses are authorized, educated, and competent to perform [14]. The CRNA describes registered nurses as autonomous healthcare professionals who practise collaboratively, provide direct healthcare services, coordinate care, support clients in managing health, and contribute across clinical practice, administration, education, and research domains [14]. The scope of practice of an individual registered nurse is shaped by foundational education, professional experience, continuing professional development, competence, client needs, employer requirements, and the practice environment [14].

In Alberta, an RN authorized by the CRNA may prescribe Schedule 1 drugs, excluding controlled drugs and substances, and order diagnostic tests within a specific clinical practice area [15]. The CRNA states that RN prescribing can support access to care, system efficiency, cost effectiveness, optimization of RN scope of practice, and innovative practice models [15]. RN prescribing is not unrestricted independent prescribing. Authorization is linked to a specific clinical practice area and requires knowledge, skill, employer support, clinical support tools, and collaborative practice relationships [15]. These requirements are consistent with the broader Canadian framework for RN prescribing [16].

The CRNA framework specifies that client needs should be stable based on assessment of acuity and predictability, and that the medications and diagnostic tests must be clearly identified in a clinical support tool [15]. Authorization requires completion of an approved nursing program for prescribing and ordering diagnostic tests, a minimum of 3000 h of RN clinical practice, and at least 750 h in the specific clinical practice area where the RN is applying for authorization [15]. CRNA competencies and guidelines further emphasize assessment, clinical judgment, prescribing accountability, diagnostic test follow-up, documentation, and escalation when presentations fall outside the RN prescriber’s authorized scope or competence [17,18].

Clinical support tools are central to the CRMC model. The CRNA requires the RN to confirm that an established clinical support tool exists in the relevant clinical practice area before authorization to prescribe Schedule 1 drugs and order diagnostic tests in that area. These tools must guide prescribing decisions and diagnostic test ordering, be developed and reviewed by an interprofessional team, be evidence-based and informed by best practices, and be reviewed at least every three years [15]. At CRMC, clinical support tools define inclusion and exclusion criteria, assessment requirements, contraindications, prescribing options, diagnostic tests, monitoring parameters, referral pathways, documentation, and follow-up expectations.

### 2.4. Description of the Triage–Treatment–Continuity Pathway

The CRMC model begins when a registered patient contacts the clinic, usually by telephone during the clinic’s telephone access hours. The first point of contact is commonly the medical office assistant (MOA). The MOA determines whether the patient is requesting a routine appointment or reporting an urgent health concern. If the patient reports symptoms that may indicate imminent death or serious adverse outcome, the MOA uses a one-page emergency recognition tool that screens for suspected myocardial infarction, suspected cerebrovascular accident or stroke, and uncontrolled active bleeding (Appendix A) [19].

The MOA emergency recognition layer is deliberately simple. MOAs do not diagnose. Their role is to recognize potential life-threatening presentations and activate the local emergency-response pathway immediately. For suspected myocardial infarction, the tool asks about pain or discomfort in the chest, light-headedness, nausea or vomiting, jaw, neck or back pain, left arm or shoulder discomfort, and shortness of breath [19,20]. For suspected cerebrovascular accident or stroke, the revised publication version uses a deliberately conservative threshold: any sudden BE-FAST feature, any single focal neurological deficit, or staff concern triggers immediate emergency escalation rather than waiting for multiple signs [21]. For active bleeding, escalation occurs when bleeding cannot be controlled by applying gauze and pressure [19].

If the emergency threshold is met, the MOA activates the emergency-response pathway according to local procedure. If the patient does not meet the immediate emergency threshold but still has an urgent, semi-urgent, unclear, or anxiety-provoking concern, the patient is escalated to the RN prescriber. This first layer protects patient safety by ensuring that clearly emergent presentations are not delayed by routine booking processes and that the RN prescriber pathway is not used as a substitute for emergency care.

The RN prescriber then performs a structured clinical assessment. The patient is classified as stable or unstable. This is the major safety decision in the pathway. Unstable patients are directed to the nearest emergency department or emergency medical services are activated. If emergency medical services are called during telephone triage, the RN prescriber remains with the patient on the telephone until emergency support arrives when feasible. Stable patients proceed to a focused history of presenting complaint and are classified using the CRMC traffic-light urgency system (Appendix B) [22].

For stable patients, the RN prescriber collects a focused history of presenting complaint, including associated symptoms, similar symptoms in contacts, provocative and palliative factors, symptom quantity and quality, severity, anatomical region and radiation, timing and duration, treatments already attempted, past medical history, and allergies. The assessment supports classification of the presentation as likely bacterial, viral, fungal, or other; consideration of specific urgent presentations such as unilateral swollen, painful, oedematous, or erythematous leg where deep vein thrombosis may need to be considered [23]; and disposition to emergency services, urgent care, same-day RN prescriber assessment, primary care provider review, pharmacist consultation, or safety-netting advice.

The traffic-light system translates clinical urgency into operational booking decisions and provides MOAs, RN prescribers, physicians, and nurse practitioners with a shared language (Appendix B) [22]. Code Red indicates a stable but urgent presentation requiring same-day assessment; it is not equivalent to a hospital emergency triage category. Code Yellow indicates a semi-urgent presentation requiring assessment within 24–48 h. Code Green indicates a non-urgent presentation usually appropriate for booking with the patient’s primary care provider within seven calendar days.

The booking algorithm is designed to match urgency with the most appropriate available clinician. Code Red patients are first matched to same-day availability with any primary care provider. If no physician or nurse practitioner appointment is available, the patient is booked with the RN prescriber on the same day when possible. If RN prescriber capacity is unavailable, the capacity issue is documented for audit and the patient is not left without disposition advice: staff seek same-day physician or nurse practitioner review where feasible, provide safety-netting, and direct the patient to urgent care, emergency department, 811, or 911 according to stability, symptoms, and clinical concern. Code Yellow patients are booked with a primary care provider today or tomorrow when available; otherwise, they are booked with the RN prescriber within the 24–48-h target where possible. If this is not possible, a safety-netted contingency disposition is documented. Code Green patients are booked with their primary care provider within seven calendar days, and any variance is documented. Patient refusal to see the RN prescriber is recorded in aggregate audit data to monitor acceptance, access barriers, and education needs without using identifiable patient information for publication [24].

### 2.5. RN Prescriber Clinical Management and Continuity

A key feature of the CRMC model is that the RN prescriber does not only triage. The RN prescriber may assess the patient by telephone, telehealth, or in person; determine whether the patient is stable; apply an approved clinical support tool; make a guided diagnosis within authorized scope; order diagnostic tests; prescribe Schedule 1 medications where appropriate, excluding controlled drugs and substances; provide health advice; give safety-netting instructions; refer to another professional; and document the encounter in the EMR. This distinguishes the model from telephone advice lines and administrative booking systems because the endpoint is not simply advice or redirection. When clinically appropriate, the endpoint is assessment and treatment within the patient’s own primary care clinic.

Diagnostic tests are ordered only when permitted by the relevant clinical support tool. The RN prescriber remains accountable for follow-up of diagnostic tests ordered under their authority, including processes for critical results, timely review when the RN prescriber is unavailable, after-hours emergency contact, and follow-up when results are not received within a reasonable time [15,18]. Prescribing occurs only after assessment and only when the medication is included in the relevant clinical support tool. The RN prescriber must use clinical judgment, consider allergies, contraindications, interactions, concurrent medications, client-specific factors, therapeutic goals, expected outcomes, adverse drug reactions, and follow-up [15,18].

The patient’s primary care provider is notified through the EMR. This preserves continuity and reduces the fragmentation that may occur when patients attend walk-in clinics, urgent care centres, or emergency departments for conditions that could be managed in primary care [25].

### 2.6. Training and Competency Framework

The CRMC model depends on RN prescribers who are specifically prepared for family medicine. The clinic developed a structured internship for RN prescribers in the family medicine setting [26]. As of January 2025, the CRMC internship runs for 18 months across five semesters and includes approximately 2940 h of specialized clinic-based preparation, which is approximately full-time-equivalent across 18 months. These hours are a clinic-level training and governance framework rather than a separate regulatory minimum. Candidates must meet CRNA authorization requirements independently, including the minimum 3000 h of RN clinical practice and at least 750 h in the specific practice area; the CRMC internship is intended to map supervised family medicine experience, clinical support tool exposure, and competence assessment onto those authorization expectations [15,26].

The early phase consolidates basic and advanced RN clinical skills in family medicine and community care. The next phase introduces supervised use of clinical support tools, with discussion of assessment and treatment plans with primary care providers. After RN prescriber licensure is obtained, the nurse gradually progresses through the full range of CRMC clinical support tools with support from senior RN prescribers, the clinical manager, the medical director, physicians, nurse practitioners, and partner community pharmacists [26].

Assessment includes theory and practice components, a high-stakes objective structured clinical examination, and demonstrated safe use of clinical support tools. Training includes common urgent and semi-urgent presentations such as upper and lower respiratory tract infections, urinary tract infections, otitis media, musculoskeletal injuries, conjunctivitis, gout, Long COVID-19, Osgood–Schlatter disease, bacterial skin infections, allergic reactions, sexually transmitted infections, diaper dermatitis, dysmenorrhoea, atopic dermatitis, gastroesophageal reflux disease, headache and migraine, herpes simplex, hormonal contraceptive care, hypertension emergency recognition, insect bites, obesity management, onychomycosis, candidiasis, post-operative hospital discharge review, shingles, tinea infections, tobacco cessation, and electrocardiogram interpretation [26].

Competence is not assumed from course completion alone. CRMC deems the RN prescriber competent to use a clinical support tool independently only after repeated correct use without recurring assistance from the medical director or other providers. This training structure is a safety and governance feature that standardizes decision-making, supports appropriate escalation, reduces variation, and increases confidence among primary care providers.

### 2.7. Data Sources, Metric Definitions, and Scenario-Based Cost-Avoidance Framework

The service evaluation counted aggregate RN prescriber pathway activity during a 12-month period from 1 April 2025 to 31 March 2026. Aggregate counts were compiled from the clinic’s EMR appointment and encounter records, RN prescriber operational pathway logs, and routine safety and complaint monitoring records. Metrics included total pathway contacts, urgency category, emergency medical services activation, emergency department referral, urgent care referral, primary care provider follow-up, patient refusal to see the RN prescriber, safety incidents, and complaints. A pathway contact was defined as one patient call, telehealth interaction, or in-person clinical encounter that entered the RN prescriber triage-treatment pathway. Telephone calls and clinical encounters were reported together because the historical operational dataset did not reliably separate modality for publication. Data were reported as aggregate service metrics, not as patient-level outcomes. Monthly totals were cross-checked against the operational log before aggregation, but no patient-level validation, chart review, external data linkage, or statistical analysis was performed.

A formal cost-effectiveness study would require additional design, patient-level data, and potentially Research Ethics Board review if patient-level or linked health-system datasets were used. This article therefore presents a scenario-based cost-avoidance framework using aggregate pathway counts and publicly available assumptions, rather than a confirmed monetary cost saving. The CIHI’s Patient Cost Estimator provides jurisdiction-level hospital cost estimates, although estimates should be interpreted carefully because of differences in care models, labour rates, and data limitations [27]. Alberta also publishes ground ambulance patient charges of CAD 250 when a patient is not transported and CAD 385 when transported; these are patient charges rather than full health-system costs [28]. Because the present evaluation did not link CRMC contacts to external ED/UCC attendance and did not identify a validated average cost for the specific types of non-admitted ED/UCC visits that might have been avoided, no average ED/UCC unit cost was substituted.

## 3. Results

### 3.1. Aggregate Service Evaluation Metrics

During the 12-month service evaluation period from 1 April 2025 to 31 March 2026, 5032 pathway contacts were managed through the RN prescriber-led pathway at CRMC. These contacts represent internal urgent and semi-urgent primary care capacity for patients who may otherwise have sought emergency department or urgent care services in the absence of timely clinic-based access. Of the 5032 contacts, 5030 were stable contacts assigned a traffic-light category and 2 were EMS/911 activations before traffic-light classification. This reconciles the denominator discrepancy in the previous table. Code Red represented 98.4% of stable traffic-light-classified contacts because the pathway primarily received pre-filtered same-day or urgent-sounding concerns rather than all clinic calls. Code Red was an operational same-day primary care category, not an emergency department acuity category; this explains why the large Code Red volume rarely translated into emergency escalation. The figure of 5032 is reported as service capacity and potential diversion, not as confirmed emergency department avoidance. Table 1 presents the aggregate service evaluation metrics for the RN prescriber-led pathway, with denominators clarified.

### 3.2. Scenario-Based Potential Emergency Department or Urgent Care Diversion

The scenario model was revised to use a consistent denominator of 5032 pathway contacts. The lowest scenario is anchored to the CIHI estimate that 15% of Canadian emergency department visits involved conditions potentially manageable in primary care [6]. This is not a validated CRMC diversion rate because the numerator and denominator differ: the CIHI begins with ED visits, whereas this evaluation begins with clinic pathway contacts. It is therefore presented only as a transparent reference point. Higher scenarios are illustrative sensitivity analyses. The 100% scenario is implausible as a real-world estimate and is retained only to show the mathematical upper bound. Table 2 presents the scenario-based potential ED/UCC diversion model using the 5032-contact denominator.

The gross cost-avoidance formula remains: gross cost avoidance = estimated avoided ED/UCC visits × average ED/UCC visit cost.

The net cost-avoidance formula remains: net cost avoidance = gross cost avoidance − RN prescriber pathway operating costs.

Operating costs should include RN prescriber salary, training costs, medical office assistant time, diagnostic testing, follow-up care, EMR support, physician or nurse practitioner consultation time, and other relevant clinic resources. Because no linked ED/UCC utilization data and no validated encounter-specific ED/UCC unit cost were available, the present manuscript reports only visit-volume scenarios and the conceptual cost formula. The estimates should be reported as potential cost avoidance, not as confirmed savings.

### 3.3. Comparison with Other Triage and Urgent-Access Models

The CRMC model differs from several established triage and urgent-access models. Emergency department triage occurs after the patient has already arrived at hospital. It prioritizes access to emergency resources but does not prevent emergency department attendance. The CRMC model intervenes before hospital attendance and creates a primary care-based alternative for stable urgent and semi-urgent patients.

Provincial or national telephone advice lines provide guidance and disposition advice. However, they may not have access to the patient’s full primary care record, may not be able to assess patients in person, may not be able to prescribe or order tests, and may not document directly into the patient’s primary care EMR. Evidence regarding remote triage is mixed; a systematic review found no statistical difference in some safety outcomes between nurse-led and general practitioner-led triage while also noting heterogeneity in utilization outcomes [29]. The CRMC model extends beyond telephone advice because the RN prescriber can assess, investigate, prescribe within scope, and arrange follow-up within the patient’s own clinic.

Walk-in clinics improve access but may fragment continuity. The patient may be assessed outside their primary care medical home, and the primary care provider may not receive timely or complete documentation. By contrast, the CRMC model maintains care within the clinic and documents the RN prescriber assessment and plan in the EMR. Nurse practitioner-led urgent care offers broader independent diagnostic and prescribing authority. The RN prescriber role does not replace nurse practitioners or physicians; instead, it offers a defined, tool-supported pathway for stable presentations within the RN prescriber’s authorized clinical practice area. Systematic review evidence suggests that nurse prescribing and RN-led primary care may contribute to care delivery and system outcomes, while also emphasizing the need for ongoing evaluation [30,31]. Table 3 summarizes the distinguishing features of the CRMC model compared with other triage and urgent-access models.

## 4. Discussion

### 4.1. Principal Findings

The CRMC RN prescriber-led model reframes triage as a clinical intervention inside primary care. Instead of using triage only to decide whether a patient should attend emergency care, urgent care, or a routine appointment, the model creates a pathway through which stable urgent and semi-urgent patients can be assessed, investigated, treated, safety-netted, referred, or escalated while remaining connected to their primary care provider.

The service evaluation demonstrates that more than 5000 pathway contacts were managed over one year during regular clinic operating and telephone access hours. This volume does not prove that 5000 emergency department visits were prevented. It does, however, demonstrate that the clinic created substantial internal urgent-access capacity for patients who may otherwise have accessed external urgent or emergency services, waited for a routine appointment, self-managed, or sought care elsewhere.

### 4.2. Safety, Access, and Continuity

The model has several safety strengths. First, it includes a simple MOA emergency recognition tool for suspected myocardial infarction, suspected stroke, and uncontrolled active bleeding (Appendix A). The revised publication version strengthens the stroke threshold by clarifying that any sudden BE-FAST sign or focal neurological deficit should trigger emergency escalation. Second, uncertain or potentially urgent presentations are escalated to the RN prescriber rather than managed solely through administrative booking. Third, the RN prescriber must classify the patient as stable or unstable. Fourth, unstable patients are referred to emergency medical services or the emergency department. Fifth, stable patients are managed only within approved clinical support tools and the RN prescriber’s competence. Sixth, care is documented in the EMR and communicated to the patient’s primary care provider.

The model also has access advantages within its operating hours. Code Red patients receive same-day attention when clinically appropriate. Code Yellow patients receive assessment within 24–48 h. Code Green patients are booked with their primary care provider within seven calendar days. This structure helps prevent urgent patients from being treated as routine because a physician schedule is full, while also protecting urgent-access capacity from being consumed by non-urgent concerns. The model should not be interpreted as replacing 24-h emergency or urgent care services.

Continuity is another important feature. Patients remain within the clinic’s EMR, and their primary care provider is notified. This may reduce fragmentation compared with walk-in clinics and may reduce duplication of assessments or investigations. The RN prescriber may also reduce patient anxiety by providing clinical interpretation, advice, treatment, and safety-netting.

### 4.3. Workforce, Practice, and Policy Implications

From a workforce perspective, the model optimizes RN prescriber scope while preserving physician and nurse practitioner capacity for complex care, chronic disease management, continuity-based care, and presentations outside RN prescriber clinical support tools. This is consistent with the CRNA framework, which recognizes RN prescribing as a mechanism to support access, system efficiency, cost effectiveness, and innovative practice models [15].

For practice, the model offers a structured way to manage urgent and semi-urgent patient concerns within the clinic. For policy, it suggests that funding models could support RN prescriber roles as part of primary care access improvement and emergency department diversion strategies in jurisdictions where RN prescribing is legally supported. For governance, replication should require employer policy, approved clinical support tools, interprofessional collaboration, RN prescriber training, medical director oversight, EMR documentation, diagnostic test follow-up processes, prescribing audit, emergency escalation pathways, and routine stakeholder feedback from patients, nurses, physicians, nurse practitioners, medical office assistants, and pharmacy partners.

### 4.4. Limitations

This article describes a single-clinic service model and reports aggregate operational data only. The findings may not be generalizable to clinics with different staffing models, patient populations, EMR systems, physician availability, RN prescriber availability, training capacity, clinic hours, or local emergency and urgent care access. The service evaluation does not include patient-level chart review, linked emergency department utilization data, patient surveys, patient interviews, clinician surveys, or formal outcome measurement. It cannot determine how many patients would definitely have attended emergency or urgent care in the absence of the model. The denominator of all registered clinic patients and total clinic call volume was not extracted, so pathway activity cannot be expressed as a rate per registered patient or as a proportion of all clinic calls.

Aggregate data cannot identify individual patient outcomes, missed diagnoses, delayed diagnoses, adverse drug events, or downstream healthcare utilization unless those events are captured through routine safety reporting. At CRMC, safety incidents would be captured through the clinic’s routine incident-reporting and clinical governance processes, and complaints would be captured through the clinic’s routine complaint process; however, aggregate monitoring may underdetect events if patients present elsewhere or do not report concerns back to the clinic. The reported absence of recorded safety incidents or complaints should therefore be interpreted as an aggregate service monitoring finding, not as proof of complete clinical safety. All authors are employees, clinicians, managers, or directors of the clinic being evaluated, and the evaluation is internal; this institutional conflict and the uniformly favourable operational findings should be considered when interpreting the results. The evidence base also includes grey literature, regulatory documents, and local operational tools, which are appropriate for a practice innovation but are not equivalent to peer-reviewed outcome studies.

### 4.5. Future Research

Future work should move beyond service evaluation. A prospective, Research Ethics Board-approved multi-site study could compare clinics with and without RN prescriber-led urgent-access pathways. Such a study could evaluate emergency department and urgent care utilization, patient outcomes, safety events, cost-effectiveness, provider workload, physician and nurse work satisfaction and quality, patient satisfaction, stakeholder feedback, continuity of care, antibiotic stewardship, diagnostic test utilization, revisit rates, and scalability. Qualitative work with patients, physicians, nurse practitioners, registered nurse prescribers, medical office assistants, pharmacists, and managers would help identify acceptability, workflow barriers, and implementation conditions for other primary care settings.

## 5. Conclusions

The CRMC Registered Nurse Prescriber-led Triage–Treatment–Continuity model offers a practical approach to urgent and semi-urgent access in family medicine. It combines medical office assistant emergency recognition, RN prescriber structured triage, stable versus unstable decision-making, traffic-light urgency classification, a booking contingency algorithm, clinical support tools, diagnostic test ordering, prescribing within scope, safety-netting, and primary care provider communication through the EMR.

During the 12-month service evaluation period from 1 April 2025 to 31 March 2026, 5032 pathway contacts were managed through the RN prescriber-led pathway. This figure demonstrates substantial internal clinic capacity for patients who may otherwise have sought emergency department or urgent care services. It should be interpreted as potential diversion, not confirmed emergency department avoidance.

The model’s main contribution is not triage alone, but the integration of triage, treatment, escalation, safety-netting, and continuity inside primary care. Further ethics-approved research is needed to evaluate patient-level outcomes, stakeholder experience, safety, comparative effectiveness, confirmed health-system utilization effects, and definitive cost-effectiveness.

## Figures and Tables

**Table 1:** Aggregate service evaluation metrics for the RN prescriber-led pathway, with denominators clarified.

Metric	Aggregate Count	Percentage/Denominator
Total RN prescriber pathway contacts	5032	100.0% of all pathway contacts
Stable contacts assigned a traffic-light code	5030	99.96% of all pathway contacts
Code Red stable same-day contacts	4950	98.4% of traffic-light-classified stable contacts
Code Yellow stable 24–48-h contacts	55	1.1% of traffic-light-classified stable contacts
Code Green stable non-urgent contacts	25	0.5% of traffic-light-classified stable contacts
EMS/911 activations before traffic-light classification	2	0.04% of all pathway contacts
Emergency department referrals after RN prescriber assessment	9	0.18% of all pathway contacts
Urgent care referrals after RN prescriber assessment	2	0.04% of all pathway contacts
Primary care provider follow-up appointments arranged after RN prescriber assessment	85	1.69% of all pathway contacts
Patient refusals to see RN prescriber	5	0.10% of all pathway contacts
Safety incidents related to triage pathway recorded in routine monitoring	0	0.00% of all pathway contacts
Complaints related to triage pathway recorded in routine monitoring	0	0.00% of all pathway contacts

**Table 2:** Scenario-based potential ED/UCC diversion model using the 5032-contact denominator.

Scenario	Assumption	Estimated Avoided ED/UCC Visits from 5032 Contacts	Approximate Frequency Equivalent
CIHI-informed reference	15% reference based on CIHI primary-care-manageable ED indicator	755	63/month; 15/week
Conservative sensitivity	25% would otherwise have attended ED/UCC	1258	105/month; 24/week
Moderate sensitivity	50% would otherwise have attended ED/UCC	2516	210/month; 48/week
High-impact sensitivity	75% would otherwise have attended ED/UCC	3774	314/month; 73/week
Upper-bound sensitivity	100% would otherwise have attended ED/UCC; implausible, illustrative only	5032	419/month; 97/week

**Table 3:** Distinguishing features of the CRMC model.

Model	Main Function	Limitation	CRMC Distinction
Emergency department triage	Prioritizes patients after ED arrival	Does not prevent ED attendance	CRMC intervenes before ED attendance
Telephone advice line	Provides advice and disposition	Usually cannot examine, prescribe, order tests, or document in PCP EMR	CRMC can move from advice to assessment and treatment
Walk-in clinic	Provides episodic access	May fragment continuity	CRMC keeps care within the medical home
Traditional family medicine booking	Books according to provider availability	Limited same-day capacity	CRMC adds RN prescriber urgent-access capacity
Nurse practitioner-led urgent care	Provides broad independent clinical management	NP availability may be limited	RN prescribers manage defined stable presentations using clinical support tools

## Data Availability

The data discussed in this manuscript consist of aggregate, non-identifying operational service metrics from Cranston Ridge Medical Clinic. No patient-level data are available for sharing. Additional aggregate information may be made available by the corresponding author upon reasonable request and subject to clinic governance, privacy requirements, and applicable legislation.

## Abbreviations

The following abbreviations are used in this manuscript:

AHA	American Heart Association
ARECCI	A Project Ethics Community Consensus Initiative
ASA	American Stroke Association
CIHI	Canadian Institute for Health Information
CNA	Canadian Nurses Association
CRMC	Cranston Ridge Medical Clinic
CRNA	College of Registered Nurses of Alberta
CST	Clinical support tool
ED	Emergency department
EMR	Electronic medical record
EMS	Emergency medical services
FTE	Full-time equivalent
MOA	Medical office assistant
NP	Nurse practitioner
PCP	Primary care provider
QI	Quality improvement
REB	Research Ethics Board
RN	Registered nurse
RN prescriber	Registered Nurse Prescriber
TCPS 2	Tri-Council Policy Statement: Ethical Conduct for Research Involving Humans
UCC	Urgent care centre

## Appendix A. Medical Office Assistant Emergency Recognition Tool

Appendix A reformats the CRMC-CSDM triaging tool used to recognize patients at risk of imminent death or serious adverse outcome. The tool is intended for trained CRMC and Cranston Smart Drug Mart staff members and supports immediate escalation through the local emergency-response process when defined thresholds or staff concern are present. In response to patient-safety review, the stroke threshold is presented using a conservative single-sign escalation rule aligned with BE-FAST messaging rather than requiring two or more signs.

**Table A1:** Medical office assistant emergency recognition tool.

Presentation Screened	Symptoms or Finding	Emergency Threshold	Action
Suspected myocardial infarction	Pain or discomfort in the chest; light-headedness, nausea, or vomiting; jaw, neck, or back pain; discomfort or pain in the left arm or shoulder; shortness of breath	Chest pain/discomfort with concerning associated symptom(s), two or more listed symptoms, or staff concern	Activate SARISS Code 2/local emergency-response process immediately
Suspected cerebrovascular accident or stroke	Sudden loss of balance; sudden blurred or changed vision; one-sided facial droop or paralysis; arm or leg weakness; slurred speech	Any one sudden BE-FAST feature, any single focal neurological deficit, or staff concern	Activate SARISS Code 2/local emergency-response process immediately
Active bleeding	Patient is actively bleeding and the bleeding cannot be stopped by applying gauze and pressure	Uncontrolled active bleeding	Activate SARISS Code 2/local emergency-response process immediately
No emergency threshold met	Patient does not meet the thresholds above	No immediate emergency threshold met	Proceed through the non-emergency clinic pathway according to urgency and scope; escalate to RN prescriber when the concern is urgent, semi-urgent, unclear, or anxiety-provoking

Note: This appendix has been reformatted and safety-clarified for publication from the original one-page operational tool. The term SARISS Code 2 refers to CRMC/CSDM local emergency-response activation. The stroke threshold has been deliberately lowered in the publication version to avoid delaying escalation of single-symptom stroke presentations.

## Appendix B. Traffic-Light System for Prioritizing Patient Bookings

Appendix B reformats the CRMC traffic-light booking tool used by medical office assistants to prioritize patient bookings into urgent, semi-urgent, and non-urgent pathways. The categories are operational scheduling labels used inside CRMC and do not replace clinical judgment, RN prescriber assessment, or emergency escalation when instability is identified. Some labels describe service streams or local booking reasons rather than diagnoses; for example, Spanish-language walk-in refers to a language-access booking stream, not a clinical condition.

**Table A2:** CRMC traffic-light system for prioritizing patient bookings.

Code	Target Timeframe	Examples of Operational Booking Categories
Red—urgent	Must be seen on the same day	Allergic reaction; anxiety; asthma/COPD; cardiac chest pain; COVID-19; dermatology (nevi); diarrhoea and vomiting; epilepsy; genitourinary medicine; hypertension management; eye infection; high infection risk; mental health over telephone; mental health visit; oncology; ophthalmology; paediatric concern; pain; trauma; urology; urinary tract infection
Yellow—semi-urgent	Must be seen within 24–48 h	Andrology; cardiology; cough, cold, and flu; dermatology; ear, nose, and throat; gastrointestinal concern; gynaecology; haematology; insomnia; complex medication review; neurology; telephone consultation; pregnancy and prenatal care; prescription refill; results; Spanish-language walk-in or interpretation-supported access; surgical concern; vascular concern; walk-in
Green—non-urgent	Must be seen within seven calendar days	Diabetes management; driver’s medical; endocrinology; family conference; forms; hospital discharge; injection; regular or follow-up medication review; meet and greet; orthopaedic concern; PAP with doctor; PAP with nurse; adult physical; paediatric physical; physiotherapy or massage referral; procedure for stitch removal; procedure for wart treatment; requisition for test or investigation; rheumatology; smoking cessation; specialist referral request; travel consultation; vaccination; WCB

Note: This appendix has been reformatted for publication from the original traffic-light booking tool. The examples are clinic-specific operational booking categories and should be adapted to local terminology, scope, staffing, and patient population before implementation elsewhere.
